# Development of a Porcine Full-Thickness Burn Hypertrophic Scar Model and Investigation of the Effects of Shikonin on Hypertrophic Scar Remediation

**DOI:** 10.3389/fphar.2018.00590

**Published:** 2018-06-05

**Authors:** Xingwang Deng, Qian Chen, Lijuan Qiang, Mingwei Chi, Nan Xie, Yinsheng Wu, Ming Yao, Dan Zhao, Jiaxiang Ma, Ning Zhang, Yan Xie

**Affiliations:** ^1^College of Clinical Medicine, Ningxia Medical University, Yinchuan, China; ^2^Department of Burns and Plastic Surgery, Xinyang Central Hospital, Xinyang, China; ^3^Medical Department, General Hospital of Ningxia Medical University, Yinchuan, China; ^4^Tissue Organ Bank & Tissue Engineering Centre, General Hospital of Ningxia Medical University, Yinchuan, China; ^5^Department of Burns and Plastic Surgery, General Hospital of Ningxia Medical University, Yinchuan, China; ^6^Department of Pathology, General Hospital of Ningxia Medical University, Yinchuan, China; ^7^Clinical Medical School, Ningxia Medical University, Yinchuan, China; ^8^School of Biomedical Sciences, Faculty of Health, Queensland University of Technology, Brisbane, QLD, Australia

**Keywords:** Shikonin, hypertrophic scars, full-thickness burn wounds, porcine hypertrophic scar models, scar remediation

## Abstract

Hypertrophic scars formed after burns remain a challenge in clinical practice. Development of effective scar therapies relies on validated animal models that mimic human hypertrophic scars. A consistent porcine full-thickness burn hypertrophic scar model has yet to be developed. We have previously reported that Shikonin induces apoptosis and reduces collagen production in hypertrophic scar fibroblasts *in vitro* and may therefore hold potential as a novel scar remediation therapy. In this study, we aimed to validate the potential of Shikonin on scar remediation *in vivo*. A novel porcine hypertrophic scar model was created after full-thickness burn wounds, and the effect of Shikonin on scar remediation was investigated. Clinical scar assessments, histology, and immunohistochemistry were used to evaluate scar appearance, morphology, and protein expression. Eight weeks after scar formation, clinical scar assessment indicated that the score of hypertrophic scars treated with Shikonin was significantly lower than that of the control group. Hypertrophic scars treated with Shikonin appeared flat, pink, and pliable. In addition, histological analysis indicated that hypertrophic scars treated with Shikonin exhibited reduced thickness of the epidermis and dermis, thin and even epithelial layers, reduced numbers of keratinocytes, uniform distribution of fibroblasts, and a parallel and loose arrangement of collagen fibers in the dermis. Moreover, immunohistochemical analysis indicated that Shikonin inhibited the expression of p63, cytokeratin 10, alpha-smooth muscle actin, transforming growth factor-beta 1, and collagen I, which play important roles in hypertrophic scar formation. Based on these results, we conclude that Shikonin has potential as a novel scar therapy.

## Introduction

Currently available therapies for hypertrophic scars are not satisfactory due to their undesirable side-effects, complex delivery routes, requirements for long-term use, and expense (Xie et al., 2015). An anti-scar therapy which is simple to use, with minimal side-effects and low cost is urgently needed. Development of effective scar therapies relies on validated animal models (Seo et al., 2013) that mimic human hypertrophic scars (Domergue et al., 2015).

Pigs have emerged as promising models to study wound healing of various wound types (Seaton et al., 2015), because they are anatomically and physiologically similar to humans (Davidson, 1998). Pigs have relatively thick epidermis, dermal papillae, and dense elastic fibers in the dermis (Lindblad, 2008). The biochemical structure of porcine collagen is similar to that of human collagen (Heinrich et al., 1971). Sullivan et al. (2001) have reported that results from a porcine study were 78% concordant with human studies, while results from small mammals and *in vitro* studies were only 53% and 57% concordant, respectively, with human studies. Numerous studies have used porcine models to evaluate the effects of myriad treatments and devices, including surgical and enzymatic debridement agents, negative pressure devices, silver dressings, collagen gel dressings, sprayed cell suspensions, and dermal substitutes (Seaton et al., 2015).

Radix arnebiae, a traditional Chinese medicine, has been clinically used to treat burns in China for thousands of years (Feng et al., 2008). Shikonin, a key ingredient of the herb Radix arnebiae, possesses various biological activities, such as anti-tumourigenic, anti-oxidant, anti-bacterial, and anti-inflammatory properties (Hsu et al., 2004). We have previously reported that Shikonin induces apoptosis and reduces collagen production in hypertrophic scar fibroblasts *in vitro* (Fan et al., 2015a,b; Xie et al., 2015). and may therefore hold potential as a novel scar remediation therapy. In the current study, we aimed to validate the potential of Shikonin for scar remediation *in vivo*. A novel porcine hypertrophic scar model was created after full-thickness burn wounds, and the effect of Shikonin on scar remediation was investigated.

## Materials and Methods

### Shikonin Solution Preparation

Shikonin powder was produced by the National Institute for the Control of Pharmaceutical and Biological Products, Beijing, China. Shikonin powder was dissolved in DMSO (Sigma-Aldrich, United States) at 10 mg/mL to create a stock solution which was stored at -20°C. The Silicone gel (Unitrump Bio, Jiangsu, China) and 0.9% sodium chloride solution (Sichuan Kelun Pharmaceutical Co., Chengdu, China) were provided by the Burn and Plastic Surgery Department of the General Hospital of Ningxia Medical University.

### Animal Studies

#### Animals

Animal studies were approved by the institutional animal ethics committee of the General Hospital of Ningxia Medical University (2015-140). Female Guangxi Bama mini-pigs, 18–20 kg and 12 weeks of age (Bainong Laboratory Animal Breeding Technology Co., Ltd., Tianjin, China, http://www.bnlab.cn/cnIndex.asp) were housed in the animal facility for two weeks to acclimatize. Animals were given a standard pellet diet and free access to water. The animals were euthanized by intravenous injection of sodium pentobarbitone (100 mg/kg, Sihuan Pharmaceutical Factory, Beijing, China) once the experiment was completed.

#### Creation of a Porcine Hypertrophic Scar Model

**FIGURE 1:**
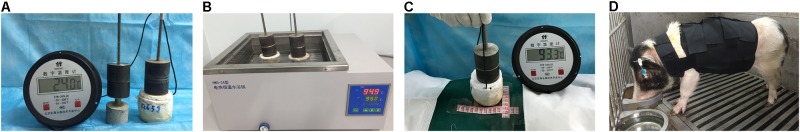
Established full-thickness burns in mini-pigs. **(A)** The homemade burn-infliction device consists of a circular aluminum block surrounded by an insulating layer of polycarbonate sheet, and a circular chunk of iron on the top. **(B)** A heating tank. The homemade burn-infliction device was pre-heated in a heating tank filled with water at 95 ± 0.2°C for 10 min. **(C)** The full-thickness dermal burn injury was created by contacting the mini-pig skin with our home-made device, with a constant pressure of 0.064 kg/cm^2^. **(D)** A homemade elasticated jacket.

Animals were fasted overnight and anesthetized with xylazine hydrochloride (0.1 mL/kg, Shengda Animal Drugs Co., Ltd., Dunhua, China) (Clover et al., 2015), Midazolam (0.1 mg/kg, Jiangsu Nhwa Pharmaceutical Co., Ltd., Xuzhou, China), and propofol (1.5 mg/kg, Jiangsu Nhwa Pharmaceutical Co., Ltd., Xuzhou, China). The flank and back hair was clipped with hair clippers, and the skin was scrubbed with a povidone iodine solution (Lekang Xiaodu Zhipin Co., Ltd., Dezhou, China) (Singer et al., 2016). The burn wound models of Singer et al. (2011) and Branski et al. (2008) were adapted for use with Guangxi Bama mini-pigs for this study. The homemade burn-infliction device consisted of a circular aluminum block (5 cm in diameter and 265 g in weight), a circular chunk of iron (1 kg weight), and an insulating layer of polycarbonate sheet (0.8 cm thick) around the aluminum block (**Figure 1A**).

**FIGURE 2:**
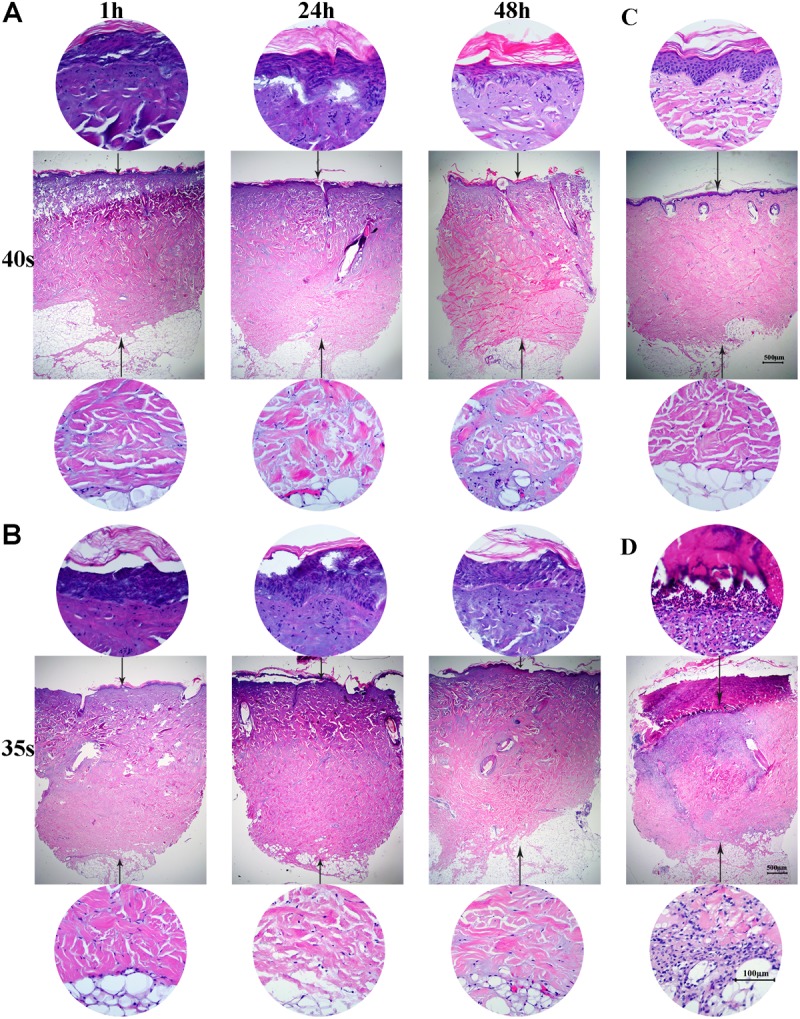
Full-thickness burns in mini-pigs. **(A)** Skin was heated for 40 s and biopsy samples were taken at 1, 24, and 48 h for histological analysis (×20 magnification). **(B)** Skin was heated for 35 s and biopsy samples were taken at 1, 24, and 48 h for histological analysis (×20 magnification). **(C)** Histological analysis of normal porcine skin. **(D)** Histological analysis of skin samples at 3 weeks after burn injury.

Wound areas were marked using a surgical marker pen, irrigated with sterile saline to ensure optimum heat coupling and burned using the heated metal device. The pig was immobilized in a lateral position on the operating table. Wounds were created on each pig’s back and flank (4 cm apart and 2 cm from the spine) using a 265-gram aluminum bar preheated in hot water at 95 ± 0.2°C for 10 min (**Figure 1B**). The heated bar was applied perpendicular to the skin surface with 1 kg of pressure (0.064 kg/cm^2^; **Figure 1C**). This burn device resulted in a circular full thickness burn wound of 5 cm in diameter. Wounds were then dressed and covered with nylon elasticated jackets to prevent animal-initiated damage (**Figure 1D**). Biopsy samples were taken at 1, 24, and 48 h after injury for histological analysis (**Figure 2A,B**). The total wound area in each pig was 3.5% of the surface area, calculated using the Meeh-Rubner formula (Swindle et al., 2012).

**FIGURE 3:**
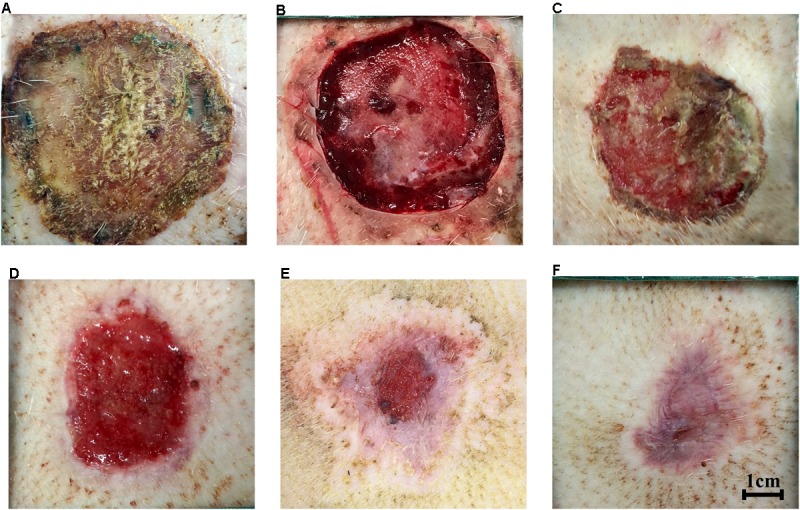
Hypertrophic scars formed after full-thickness burns in mini-pigs. **(A)** Eschar formation at 3 weeks after burn injury. **(B)** Clearance of necrotic eschar at 3 weeks after burn injury. **(C)** Necrotic tissue autolysis at 4 weeks after burn injury. **(D)** Granulation tissue formation at 5 weeks after burn injury. **(E)** Wound contraction and re-epithelialization at 6 weeks after burn injury. **(F)** Hypertrophic scar formation after full-thickness burn wounds closed at 7 weeks after burn injury.

At 21 days after injury, debridement surgeries were conducted to promote the formation of hypertrophic scars (**Figure 3A**). The center of necrotic eschar was cleared and a 5-mm-wide necrotic band was retained (**Figure 3B**). Wound necrotic tissue gradually dissolved, fresh granulation tissue developed and the wound began to epithelialize (**Figures 3C–E**). By 7 weeks, the full-thickness burn wound had closed and a hypertrophic scar was formed (**Figure 3F**). Topical sterile dressings underneath the nylon jacket were used to protect the wounds. Velcro and elastic bands were used to fix the jackets around the mini-pigs. Cefoperazone (50 mg/kg, Zhongnuo Pharmaceutical Co., Ltd., Shijiazhuang, China) was injected intramuscularly to prevent infection during surgery. Analeptics (0.1 mL/kg, Shengda animal drugs Co., Ltd., Dunhua city, China) were then intravenously injected. Finally, animals were transferred to individual cages and monitored closely for adverse outcomes during their post-procedure recovery. Post-surgical pain was managed with oral analgesia.

### Effects of Shikonin on Hypertrophic Scar Remediation

A total of 27 hypertrophic scars were created and analyzed for this study. There were three treatment groups: the positive control group was treated with Silicone gel, the negative control group was treated with saline and the Shikonin group was treated with Shikonin. In the Silicone gel and saline groups, 0.3 mL of Silicone gel (Unitrump Bio) or 1 mL of 0.9% sodium chloride solution (Kelun Pharmaceutical Co., Ltd.) were applied to the surface of the scars every 2 days. In the Shikonin group, 1 mL of 1.0 μg/mL Shikonin (National Institute for the Control of Pharmaceutical and Biological Products) was sprayed onto the surface of hypertrophic scars every 2 days. Hypertrophic scars were then covered with sterile gauze, bandaged with medical cotton pads, and covered with elasticated jackets. Hypertrophic scar area, clinical scar assessment, the thickness of epidermis and dermis, dermal thickness reduction, and protein expression were measured to compare the therapeutic effects of the different treatments.

#### Clinical Scar Assessments

Clinical scar assessments including color, height, texture, and final outcome were judged based on the MAPS (Matching Assessment of Photographs and Scars), Vancouver Scar Scales and Hamilton scar scales with modifications (Crowe et al., 1998; Wang et al., 2008; Tyack et al., 2012). The wound color was rated from 0 to 5, with 0 = normal skin color, 1 = partial normal color, 2 = pink, 3 = red, 4 = purple, and 5 = dark purple. The scar height was rated from -1 to 5, with -1 = depressed, 0 = not raised, 1 = 1 mm raised, 2 = 2 mm raised, 3 = 3 mm raised, 4 = 4 mm raised, and 5 ≥ 5 mm raised. Scar texture was rated from 0 to 5, with 0 = normal, 1 = very soft, 2 = soft, 3 = slightly hard, 4 = moderately hard, and 5 = very hard. The final outcome was rated from 0 to 5 and was based on the general impression of the scar (color, hair, contraction, surface, border, height), with 0 = normal skin, 1 = minor scar, 2 = minor–moderate scar, 3 = moderate scar, 4 = moderate–severe scar, and 5 = severe scar.

Hypertrophic scar areas were photographed and quantified from images taken using Image Pro Plus v6.0 software (Media Cybernetics, Rockville, MD, USA).

#### Histology

Specimens were obtained using a 6-mm biopsy punch from wounds at 1, 24, and 48 h, and 21 days post-burn, and from hypertrophic scars at weeks 0, 1, 4, and 8. Samples were fixed in 10% neutral buffered formalin for 24 h and embedded in paraffin. Paraffin sections of 4 μm thickness were stained with H&E (Sigma-Aldrich, Steinheim, Germany). Quantitative measurements of the epidermal thickness and dermal thickness were calculated across the entire section of H&E-stained images (Clover et al., 2015) from five measurements per image (Olympus CX31, Tokyo, Japan). The epidermal thickness reduction rate was calculated using the formula: (original epidermal thickness - epidermal thickness after treatment)/original epidermal thickness × 100%). The dermal thickness reduction rate was calculated using the formula: (original dermal thickness - dermal thickness after treatment)/original dermal thickness × 100%).

#### Immunohistochemistry

Immunohistochemical analysis was conducted on porcine hypertrophic scar tissues (Shin et al., 2016). Paraffin sections of 3 μm thickness were stained for cytokeratin 10 (CK10) (Abcam, Cambridge, United Kingdom), p63 (Biocare Medical, Concord, CA, United States), transforming growth factor-beta 1 (TGF-β1) (Abcam, Cambridge, United Kingdom), alpha-smooth muscle actin (α-SMA) (Biocare Medical, Concord, CA, United States) and collagen I (Epitomics, Burlingame, Concord, CA, United States). Slides were dewaxed, hydrated and washed. Sections were treated with citrate buffer (0.001 M, pH 6.0) at 105°C for 2 min. All slides underwent an endogenous peroxide removal step (3%H_2_O_2_, 10 min) followed by blocking with 5% bovine serum albumin for 20 min at 25°C. The primary antibodies p63 (1:200), collagen I (1:100), and α-SMA (1:150) were incubated for 2 h at 25°C. Antibodies against CK10 (ab111447, 1:200) and TGF-β1 (ab92486, 1:150) were incubated overnight at 4°C. After washing with PBS (Gibco; Thermo-Fisher Scientific, Suzhou, China), polymer helper was added and incubated for 20 min at 25°C, followed by adding polyperoxidase anti-mouse/rabbit-IgG (OriGene Technologies, Inc., Beijing, China) for 30 min at 25°C. Finally, 0.05% 3,3-diamino-benzidine (Sigma, St Louis, MO, United States) was added and incubated followed by counterstaining with hematoxylin.

Image-Pro plus 6.0 software (Media Cybernetics) was employed to quantify the immunohistochemical staining of protein expression, as previously described (Chen et al., 2013). Briefly, all images (200×) were captured with an upright light microscope and camera (Olympus CX31). Five random images from each sample were delineated to measure the IOD of positive staining.

The reduction rate of protein (p63, CK10, α-SMA, TGF-β1 and collagen I) expression was calculated using the formula: (original IOD - IOD after treatment)/original IOD × 100%.

### Statistical Analysis

All data were analyzed using SPSS 18.0 software (IBM Co., Chicago, IL, United States). All experiments were performed in triplicate, with each treatment tested individually three times on three separate female mini-pigs; and data were expressed as mean ± standard error. One-way ANOVA and Tukey’s *post hoc* test were used to identify statistically significant differences between the treatment groups, with significance indicated by *P* < 0.05.

## Results

### Hypertrophic Scars Formed After Full-Thickness Burns in Mini-Pigs

A full-thickness dermal burn injury was created by contacting the mini-pig skin with our home-made device (**Figures 1A–C**). Wound dressings for pigs were designed by us and made by a tailor (**Figure 1D**). Biopsies were taken from burn wounds at 1, 24 and 48 h, and were examined histologically to assess the depth of the thermal injury (**Figures 2A,B**). For the skin heated for 40 s, a full-thickness burn wound was observed after 24 h and the damage extended over the bottom layer of dermis at 48 h (**Figure 2A**), while for the skin heated for 35 s, a full-thickness burn wound was observed after 48 h and the depth of the wound was 4012.15 ± 102.5 μm (**Figure 2B**). Normal porcine skin was used as control (**Figure 2C**). Based on these results, 35 s was chosen to create the full-thickness dermal burn injury. Three weeks after injury, full-thickness necrotic eschar had formed (**Figures 2D**, **3A**) and debridement surgeries were conducted to promote the formation of hypertrophic scars. The center of necrotic eschar was cleared and a 5-mm-wide necrotic band was retained (**Figure 3B**). Four weeks after injury, necrotic tissue autolysis was observed (**Figure 3C**). At 5 weeks, granulation tissue has formed (**Figure 3D**). At 6 weeks, wound contraction and re-epithelialization were observed (**Figure 3E**). By 7 weeks, the full-thickness burn wound had closed and hypertrophic scars were formed (**Figure 3F**).

**FIGURE 4:**
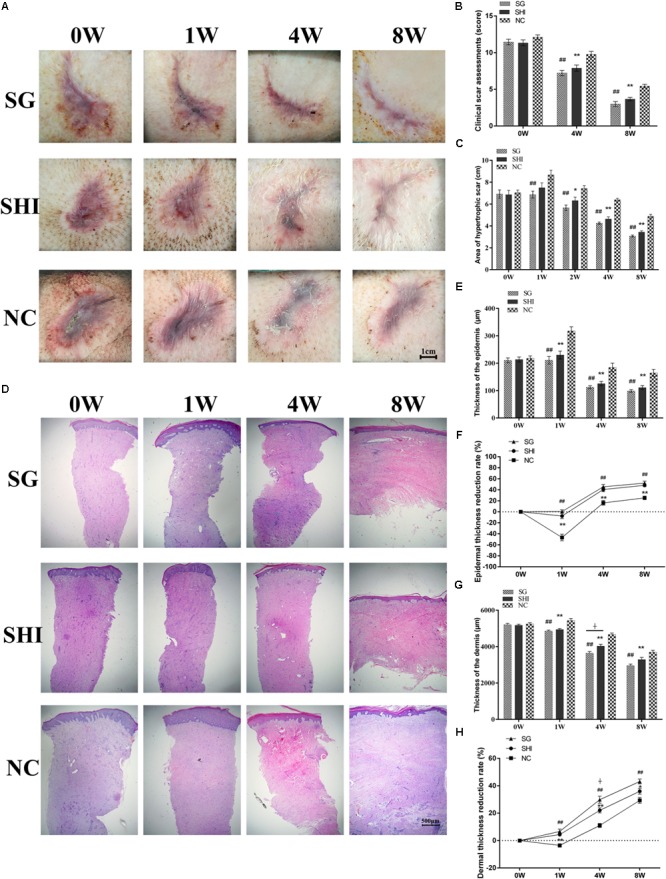
Effects of Shikonin on hypertrophic scar remediation. **(A)** Holism and gross appearance of hypertrophic scars. Porcine hypertrophic scars were treated with Silicone gel (SG), Shikonin (SHI) or saline (NC) for 1, 4, and 8 weeks. **(B)** Clinical scar assessments. Clinical scar assessments were judged based on the MAPS and Hamilton scar scales with modifications. **(C)** Quantification of hypertrophic scar area. **(D)** Histological analysis of hypertrophic scars (×20 magnification). **(E)** Quantification of epidermal thickness. **(F)** Quantification of epidermal thickness reduction. **(G)** Quantification of dermal thicknes. **(H)** Quantification of dermal thickness reduction. Significance of the differences between SHI and NC were set at ^∗^*P* < 0.05 and ^∗∗^*P* < 0.01. Significance of the differences between SG and NC were set at ^#^*P* < 0.05 and ^##^*P* < 0.01. Significance of the differences between SHI and SG were set at ^+^*P* < 0.05.

Hypertrophic scars appear dark purple, raised, firm, and congested with no hair (**Figure 4A**). The area and the thickness of hypertrophic scars were quantified. The initial scar area was 6.93 ± 0.34 cm^2^. The average thickness of the initial epidermis and dermis were 214 ± 8.94 μm and 5207.27 ± 64.71 μm, respectively, while the epidermal thickness and dermal thickness of normal skin are 42.15 ± 2.5 μm and 2831.52 ± 40.41 μm, respectively.

### Effects of Shikonin on Hypertrophic Scar Remediation

#### Clinical Scar Assessment of Porcine Hypertrophic Scar Remediation

Porcine hypertrophic scars were treated with Shikonin, Silicone gel or saline. Holism and gross appearance of hypertrophic scars were evaluated and photographed (**Figure 4A**). The initial hypertrophic scars exhibited height, congestion and purple coloration, and hard texture at week 0. Four weeks later, hypertrophic scars treated with Shikonin or Silicone showed a smaller scar area, lower height, contraction, red color, and hard texture, compared with the scars treated with saline. By 8 weeks, hypertrophic scars treated with Shikonin or Silicone gel appeared flattened and less contracted, pink in color, pigmented, and hard in texture, compared with the scars treated with saline. Clinical scar assessment, which is based on scar tissue color, raised height, hardness and final outcome, indicated that the hypertrophic scars treated with Shikonin or Silicone gel were significantly lower than the negative control saline group at 4 and 8 weeks (*P* < 0.01, **Figure 4B**).

#### Reduced Area of the Hypertrophic Scars Treated With Shikonin

The areas of the hypertrophic scars treated with Shikonin, Silicone gel and saline were quantified (**Figure 4A**). The initial area of the hypertrophic scars treated with Shikonin was 6.86 ± 0.39 cm^2^ at week 0. The area increased to 7.50 ± 0.43 cm^2^ at week 1, then gradually decreased to 6.32 ± 0.32 cm^2^ at week 2, 4.63 ± 0.20 cm^2^ at week 4 and 3.43 ± 0.12 cm^2^ at week 8. The initial area of the hypertrophic scars treated with Silicone gel was 6.89 ± 0.39 cm^2^ at week 0. The area increased to 6.86 ± 0.32 cm^2^ at week 1, then gradually decreased to 5.66 ± 0.24 cm^2^ at week 2, 4.25 ± 0.10 cm^2^ at week 4 and 3.07 ± 0.09 cm^2^ at week 8. The initial area of the hypertrophic scars treated with saline was 7.05 ± 0.24 cm^2^ at week 0. The area increased to 8.67 ± 0.42 cm^2^ at week 1, then gradually decreased to 7.43 ± 0.26 cm^2^ at week 2, 6.40 ± 0.13 cm^2^ at week 4, and 4.88 ± 0.17 cm^2^ at week 8. The area of the hypertrophic scars treated with Shikonin or Silicone gel at weeks 2, 4, and 8 was significantly less than that of the scars treated with saline (*P* < 0.05, **Figure 4C**). There was no significant difference between the area of the scars treated with Shikonin and those treated with Silicone gel at any of the time-points evaluated.

#### Histological Characteristics of Hypertrophic Scars Treated With Shikonin

Epithelial morphology of hypertrophic scars was analyzed using histology (**Figure 4D**). One week after scar formation, hypertrophic scars treated with Shikonin, Silicone gel or saline all showed increased epithelial thickness, uneven thickness, and dense epithelial cells, compared with the initial hypertrophic scars at week 0. At weeks 4 and 8, decreased epithelial thickness and epithelial cell density were observed in all three groups. At week 8, hypertrophic scars treated with Shikonin or Silicone gel showed thin and uniform thickness of the epidermis and reduced numbers of epithelial cells, compared with the scars treated with saline (**Figure 4D**).

The epithelial thickness of hypertrophic scars treated with Shikonin, Silicone gel or saline was quantified (**Figure 4D**). The initial epidermal thickness of the hypertrophic scars treated with Shikonin was 213.71 ± 9.30 μm at week 0. The thickness increased to 229.91 ± 14.31 μm at week 1, then gradually decreased to 125.66 ± 8.14 μm at week 4, and to 111.02 ± 7.14 μm at week 8. The initial epidermal thickness of the hypertrophic scars treated with Silicone gel was 210.45 ± 8.79 μm at week 0. The thickness increased to 210.736 ± 13.99 μm at week 1, then gradually decreased to 112.28 ± 5.66 μm at week 4, and to 98.71 ± 4.86 μm at week 8. The initial epidermal thickness of the hypertrophic scars treated with saline was 217.85 ± 8.73 μm at week 0. The thickness increased to 318.04 ± 14.99 μm at week 1, then gradually decreased to 184.65 ± 15.79 μm at week 4, and to 164.61 ± 12.97 μm at week 8. The epidermal thickness of the hypertrophic scars treated with Shikonin or Silicone gel at weeks 1, 4, and 8 was significantly less than that of the scars treated with saline (*P* < 0.01, **Figures 4E,F**). There was no significant difference between the epidermal thickness of the hypertrophic scars treated with Shikonin and those treated with Silicone gel at week 1, 4, and 8.

The dermal thickness of hypertrophic scars treated with Shikonin, Silicone gel or saline was quantified (**Figure 4D**). The initial dermal thickness of hypertrophic scars treated with Shikonin was 5170.8 ± 56.40 μm at week 0. The thickness gradually decreased to 4940.63 ± 53.75 μm at week 1, 4028.36 ± 99.31 μm at week 4, and 3361.99 ± 127.20 μm at week 8. The initial dermal thickness of the hypertrophic scars treated with Silicone gel was 5203.38 ± 70.88 μm at week 0. The thickness gradually decreased to 4848.66 ± 62.61 μm at week 1, 3636.73 ± 92.84 μm at week 4, and 2954.87 ± 83.90 μm at week 8. The initial dermal thickness of the hypertrophic scars treated with saline was 5247.65 ± 66.85 μm at week 0. The thickness increased to 5433.48 ± 110.70 μm at week 1, and gradually decreased to 4666.42 ± 77.38 μm at week 4, and to 3700.67 ± 100.09 μm at week 8. The dermal thickness of hypertrophic scars treated with Shikonin or Silicone gel at weeks 1, 4 and 8 was significantly less than that of those treated with saline (*P* < 0.01, **Figures 4G,H**). There was no significant difference in the epidermal thickness of hypertrophic scars treated with Shikonin or Silicone gel at weeks 1 and 8.

**FIGURE 5:**
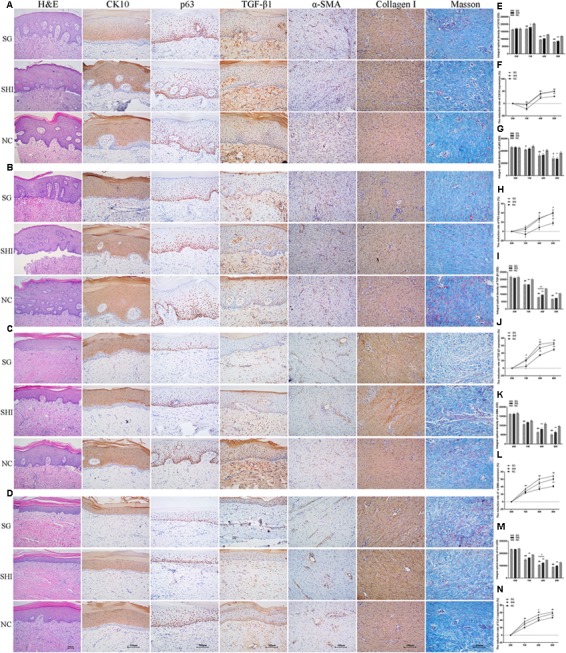
Effects of Shikonin on protein expression in porcine hypertrophic scar models. Immunohistochemistry was performed to detect the expression of specific markers in hypertrophic scar tissues, including CK10, p63, TGF-β1, α-SMA, and collagen I. **(A)** Immunohistochemical analysis of CK10, p63, TGF-β1, α-SMA, and collagen I in hypertrophic scars at week 0 (×200 magnification). **(B)** Immunohistochemical analysis of hypertrophic scars at week 1. **(C)** Immunohistochemical analysis of hypertrophic scars at week 4. **(D)** Immunohistochemical analysis of hypertrophic scars at week 8. **(E)** Quantification of CK10 staining. **(F)** Quantification of the reduction rate of CK10 staining. **(G)** Quantification of p63 staining. **(H)** Quantification of the reduction rate of p63 staining. **(I)** Quantification of TGF-β1 staining. **(J)** Quantification of the reduction rate of TGF-β1 staining. **(K)** Quantification of α-SMA staining. **(L)** Quantification of the reduction rate of α-SMA staining. **(M)** Quantification of collagen I staining. **(N)** Quantification of the reduction rate of collagen I staining. Significance of the differences between SHI and NC were set at ^∗^*P* < 0.05 and ^∗∗^*P* < 0.01. Significance of the differences between SG and NC were set at ^#^*P* < 0.05 and ^##^*P* < 0.01. Significance of the differences between SHI and SG were set at ^+^*P* < 0.05 and ^++^*P* < 0.01.

The collagen morphology of hypertrophic scars was analyzed by Masson’s trichrome staining. The initial hypertrophic scar showed dense, disordered collagen fibers and large numbers of fibroblasts. At week 8, hypertrophic scars treated with Shikonin or Silicone gel showed loose, parallel bundles of collagen fibers and decreased numbers of fibroblasts in the dermis, compared with the scars treated with saline (**Figures 5A–D**). Taken together, these data indicated that Shikonin reduced the scar area, decreased the epidermal and dermal thickness and improved the final outcome of the hypertrophic scars.

#### Effects of Shikonin on Protein Expression in a Porcine Hypertrophic Scar Model

Immunohistochemistry was performed to detect the expression of specific markers in hypertrophic scar tissues, including cytokeratin 10 (CK10), p63, transforming growth factor-beta 1 (TGF-β1), alpha-smooth muscle actin (α-SMA) and collagen I.

CK10, a differentiation marker, is mainly expressed in the cytoplasm of differentiated keratinocytes (Ota et al., 2014). At week 0, strong positive staining of CK10 was detected in the epidermis of hypertrophic scars. At week 1, positive staining of CK10 in hypertrophic scars treated with Shikonin, Silicone gel, or saline was enhanced compared with week 0. At weeks 4 and 8, positive staining of CK10 in all three groups was weaker compared with week 0 (**Figures 5C,D**). Positive staining of CK10 in hypertrophic scars treated with saline at weeks 1, 4, and 8 was significantly stronger than that of the hypertrophic scar treated with Shikonin or Silicone gel (*P* < 0.01, **Figures 5E,F**). There was no significant difference in the positive staining of CK10 between hypertrophic scars treated with Shikonin and those treated with Silicone gel at weeks 1, 4 and 8 (*P* > 0.05). Shikonin gradually decreased CK10 expression in hypertrophic scars.

The proliferation marker p63 is mainly expressed in the nucleus of proliferative keratinocytes (Yang et al., 1999). At week 0, strong positive staining of p63 was detected in the epidermis of hypertrophic scars. At week 1, positive staining of p63 in hypertrophic scars treated with saline was enhanced, compared with week 0. At weeks 4 and 8, positive staining of p63 was observed in hypertrophic scars treated with Shikonin or Silicone gel, while staining of p63 was strongly positive in the saline group (**Figures 5C,D**). The p63 staining of the hypertrophic scars treated with saline at weeks 4 and 8 was significantly stronger than that of the hypertrophic scars treated with Shikonin or Silicone gel (*P* < 0.05, **Figures 5G,H**). There was no significant difference in p63 staining between hypertrophic scars treated with Shikonin and those treated with Silicone gel, at weeks 1, 4, and 8 (*P* > 0.05). Shikonin gradually decreased p63 expression in hypertrophic scars.

TGF-β1, a multifunctional cytokine which is present in the cytoplasm and intercellular stroma, and is mainly distributed in the epithelial layer of cells, especially basal cells and dermal papilla fibroblasts, is believed to have the closest relationship with scar formation (Cuttle et al., 2006). At week 0, strong positive staining of TGF-β1 was detected in the dermis of hypertrophic scars. At weeks 1, 4, and 8, positive staining of TGF-β1 gradually weakened in all three treatment groups (**Figures 5B–D**). The positive staining of TGF-β1 in hypertrophic scars treated with Shikonin or Silicone gel at weeks 1, 4, and 8 was significantly lower than that of the hypertrophic scars treated with saline (*P* < 0.01, **Figures 5I,J**). There was no significant difference in positive staining of TGF-β1 between hypertrophic scars treated with Shikonin and those treated with Silicone gel at weeks 1 and 8 (*P* > 0.05). Shikonin gradually decreased TGF-β1 expression in hypertrophic scars.

α-SMA, a marker of myofibroblasts, was detected in fibroblasts and vascular endothelial cells (Carthy et al., 2015). At week 0, strongly-positive staining of α-SMA was detected in the dermis of hypertrophic scars. At weeks 1, 4, and 8, positive staining of α-SMA gradually weakened in all three treatment groups, compared with week 0 (**Figures 5B–D**). The positive staining of α-SMA in hypertrophic scars treated with Shikonin and Silicone gel at weeks 4 and 8 was significantly lower than in the saline group (*P* < 0.01, **Figures 5K,L**). There was no significant difference in the positive staining of α-SMA between hypertrophic scars treated with Shikonin and those treated with Silicone gel at weeks 1, 4, and 8 (*P* > 0.05). Shikonin gradually decreased α-SMA expression in hypertrophic scars.

Collagen type I secreted by fibroblasts is the most abundant collagen of the human body, and is present in scar tissue (Xue and Jackson, 2015). At week 0, strong positive staining of collagen I was detected in the dermis of hypertrophic scar tissues. At weeks 1, 4, and 8, positive staining of collagen I gradually weakened in all three treatment groups, compared with week 0 (**Figures 5B–D**). Positive staining of collagen I in hypertrophic scars treated with saline at weeks 1, 4, and 8 was significantly higher than that of the hypertrophic scars treated with Shikonin or Silicone gel (*P* < 0.01, **Figures 5M,N**). There was no significant difference in the positive staining of collagen I between hypertrophic scars treated with Shikonin and those treated with Silicone gel at weeks 1 and 8 (*P* > 0.05). Shikonin caused a gradual decrease in collagen I expression in hypertrophic scars.

Taken together, these results of immunohistochemical analysis demonstrated that Shikonin gradually decreased the expression of p63, CK10, α-SMA, TGF-β1, and collagen I in hypertrophic scars.

## Discussion

Hypertrophic scars formed after burns remain a challenge in clinical practice (Kravez et al., 2017). The classic scar treatment, surgery, has been used to release contracture after a scar is mature (Hayashida and Akita, 2017); however, early scar intervention is important for scar remediation. Non-surgical therapies for hypertrophic scars include pressure garments, laser treatment, cryotherapy, radiation, intralesional corticosteroid administration, and Silicone gel sheets (Friedstat and Hultman, 2014). However current scar treatments are not satisfactory because they are time-consuming or expensive (Finnerty et al., 2016); therefore, the development of novel hypertrophic scar treatments is necessary.

Pre-clinical animal models are of great importance for studying the development of hypertrophic scars and evaluating the therapeutic effects of anti-scar treatment. Several models of hypertrophic scarring have been reported. Full-thickness burns were incurred by pressing the end of a pre-heated (≥95°C) brass block against the rat’s dorsum for 10 s (Kravez et al., 2017). The most frequently used hypertrophic scar model is the rabbit ear excisional wound model, which does not reflect burn injuries (Domergue et al., 2015). Another scar model was created by implanting human hypertrophic scar tissue into immunodeficient mice (Kischer et al., 1989). Cuttle et al. (2006) reported a porcine deep dermal partial thickness burn model with hypertrophic scarring created by applying a glass bottle containing water at 92°C to the skin of a juvenile large white pig for 14 s. Singer et al. (2009, 2012) developed a porcine partial thickness burn model with hypertrophic scarring created by applying an aluminum bar preheated to 80°C to domestic pig skin for 20 s . Due to their low cost, domestic pigs have been widely used to create hypertrophic scar models as mentioned above (Cuttle et al., 2006; Singer et al., 2009, 2012); however, rapid growth of their body shape during the experiment affects the experimental results (Zhu et al., 2007). In our study, Bama miniature pigs (4 months old, weighing 18–20 kg) were used. The Bama miniature pig, a unique small-scale pig, which is characterized by its small size, slow growth, and white skin, is more convenient for surgery, shows good genetic stability and small individual differences, and is consequently a better experimental animal for pharmacological studies (Chandraratna, 1996; Ramos et al., 2008; Yu et al., 2014). Studies have shown that skin stratification, thickness, and structure of the Bama miniature pig are similar to those of human skin (Chen et al., 2006). We have observed that the average length of the piglet chest wall increased by 2.76 ± 0.33 cm in 5 months.

Full-thickness burn wounds and delayed re-epithelialization are the most important factors affecting the formation of hypertrophic scars (Domergue et al., 2015). In the current study, we established a reproducible burn hypertrophic scar model using the Bama miniature pig by applying a homemade heating device for 35 s followed by debridement surgery. A burn device with a fixed pressure and insulation layer were designed and heated at 95°C for 35 s to create a uniform burn wound. By 48 h after creation of the burn, the damage had progressed and extended to the full layer of the dermis as shown by H&E staining (**Figure 2B**). This progression of burn injury after initial heat damage is also seen in burn patients (Nanney et al., 1996). At week 3 after burn creation, delayed surgical debridement was conducted to promote formation of hypertrophic scars (**Figure 3B**). Seven weeks after burn injury, the full-thickness burn wound had closed and a hypertrophic scar formed (**Figure 3F**). In our experiment, the total area of scald in each piglet accounted for 3.5% of the total surface area. Our hypertrophic scars were characterized by increased thickness of the epidermis and dermis, dense proliferating fibroblasts, excessive collagen deposition, and strong positive staining of CK10, p63, α-SMA, TGF-β1 and collagen I, which is similar to previous reports of porcine hypertrophic scars (Cuttle et al., 2006; Gurtner et al., 2011; Singer et al., 2016). Macroscopic, histologic, and biologic criteria were similar to human hypertrophic scars (Domergue et al., 2015; Zhang et al., 2017).

The area of our porcine hypertrophic scars formed after creation of full-thickness burn wounds, which was 6.93 ± 0.34 cm^2^, is larger than others reported in previous studies (Singer et al., 2009, 2012). A large area of scar provides a better view for observing the effects of anti-scar therapies. Our hypertrophic scar model has provided a useful tool to test various promising therapies for hypertrophic scars and to observe the processes of hypertrophic scar remediation. This also allows us to further study and understand the mechanism of healing and scarring remediation of full-thickness burn wounds in humans.

Radix Arnebiae, a perennial herb, has been clinically used to treat burns and manage scars for thousands of years (Tang, 2010). Shikonin (C16H16O5), a natural naphthoquinone extracted from Radix Arnebiae, has been widely demonstrated to possess various biological activities, such as anti-inflammatory, anti-bacterial, anti-angiogenic, and anti-tumorigenic properties (Hashimoto et al., 1999; Chen et al., 2002; Gao et al., 2002; Hsu et al., 2004; Chang et al., 2010). We have previously reported that Shikonin inhibits cell proliferation, induces apoptosis and reduces collagen production *in vitro*; and may therefore hold potential as a novel scar remediation therapy (Fan et al., 2015a,b; Xie et al., 2015). In the current study, we aimed to validate the potential of Shikonin for scar remediation *in vivo*. In this study, a porcine hypertrophic scar model was created and treated with Shikonin, positive control Silicone gel or negative control saline for 8 weeks. Silicone gels have been widely used in the treatment of hypertrophic scars (van der Wal et al., 2010). Clinical scar assessment indicated that the scores of hypertrophic scars treated with Shikonin or Silicone gel were significantly lower than those of the saline group (**Figure 4B**). Hypertrophic scars treated with Shikonin or Silicone gel appeared flat, pink, and pliable. The area of hypertrophic scars treated with Shikonin or Silicone gel was significantly less than that of the saline group at week 8 (**Figure 4C**).

Histological analysis indicated that hypertrophic scars treated with Shikonin exhibited reduced thickness of the epidermis and dermis, a thin and even epithelial layer, reduced numbers of keratinocytes, uniform distribution of fibroblasts, and loose, parallel bundles of collagen fibers at week 8, compared with the initial hypertrophic scar at week 0. The epithelial and dermal layer thickness of hypertrophic scars treated with Shikonin or Silicone gel was significantly less than that of the saline group (**Figures 4E,G**).

The epidermis partitions into two layers: the proliferative layer stained with p63 and the differentiated layer stained with CK10. The CK10 and p63 staining of hypertrophic scars treated with Shikonin or Silicone gel were weaker than in the saline group at week 8 (**Figures 5E,G**). In addition, staining of TGF-β1, α-SMA and collagen I in hypertrophic scars treated with Shikonin or Silicone gel were only weakly positive compared with the saline group (**Figure 5D**). TGF-β1 is the main profibrotic cytokine in most fibrotic pathways (Kwan and Tredget, 2017; Zhao et al., 2017). The TGF-β1 staining of the hypertrophic scars treated with Shikonin or Silicone gel was significantly lower than that of the hypertrophic scars treated with saline at week 8 (**Figures 5I,J**). Our data indicate that Shikonin may inhibit fibrosis by targeting the TGF-β1 signaling pathway (Zhang et al., 2016; Zhao et al., 2017) and therefore remediate hypertrophic scars. Myofibroblasts are characterized by the presence of α-SMA (Franz et al., 2009). Fibroblasts can be activated to differentiate into myofibroblasts by TGF-β1 (Zhao et al., 2017). The α-SMA staining of the hypertrophic scars treated with Shikonin or Silicone gel at week 8 was significantly lower than that of hypertrophic scars treated with saline (**Figures 5K,L**). Our data indicate that Shikonin reduces α-SMA expression and therefore inhibits contracture deformity in hypertrophic scars. Collagen is one of the key components of the extracellular matrix. Collagen I and collagen III are the two major collagen types in human skin (Sullivan et al., 2001). The collagen I staining of the hypertrophic scars treated with saline at week 8 was significantly higher than that of the hypertrophic scars treated with Shikonin or Silicone gel (**Figures 5M,N**). We previously found that Shikonin reduced the expression of collagen I genes to attenuate collagen synthesis (Xie et al., 2015). Our pig study confirmed that Shikonin reduces collagen I expression, and therefore improves hypertrophic scar texture and pliability.

While our preclinical research (*in vitro* and *in vivo*) answered basic questions about Shikonin in scarring, it is unknown how it will interact with the human body. Proof-of-concept/validation trials in humans will be conducted to further validate the effects of Shikonin on human scar amelioration. As a Phase I clinical trial launches, we will answer more research questions concerning how Shikonin acts on human skin, the side effects associated with increased dosage, and the best method of administration way to restrict risks and maximize benefits.

In summary, we have demonstrated that Shikonin remediated hypertrophic scarring by reducing the thickness of the epidermis and dermis, reducing the amount of collagen produced and inhibiting the expression of p63, CK10, α-SMA, TGF-β1 and collagen I, using a porcine model of hypertrophic scarring. Therefore, we conclude that Shikonin has potential as a novel scar therapy.

## Author Contributions

YX and YW: study conception and design. XD, QC, LQ, DZ, and JM: acquisition of data. MC, NX, YW, MY, and NZ: analysis and interpretation of data. XD and YX: drafting of manuscript. YX: critical revision.

## Conflict of Interest Statement

The authors declare that the research was conducted in the absence of any commercial or financial relationships that could be construed as a potential conflict of interest.

## Abbreviations

ANOVA: analysis of variance
DMSO: dimethyl sulfoxide
H&E: Hematoxylin and Eosin
IOD: integrated optical density
MAPS: Matching Assessment of Photographs and Scars
PBS: phosphate-buffered saline.
